# Engineered heparins as new anticoagulant drugs

**DOI:** 10.1002/btm2.10042

**Published:** 2016-11-21

**Authors:** Deepika Vaidyanathan, Asher Williams, Jonathan S. Dordick, Mattheos A.G. Koffas, Robert J. Linhardt

**Affiliations:** ^1^ Dept. of Biology Rensselaer Polytechnic Institute Troy NY 12180; ^2^ Dept. of Chemical and Biological Engineering Rensselaer Polytechnic Institute Troy NY 12180; ^3^ Dept. of Biomedical Engineering Rensselaer Polytechnic Institute Troy NY 12180; ^4^ Dept. of Chemistry and Chemical Biology Center for Biotechnology and Interdisciplinary Studies, Rensselaer Polytechnic Institute Troy NY 12180

**Keywords:** bioengineered, chemoenzymatic synthesis, glycosaminoglycans, metabolic engineering

## Abstract

Heparin is an anionic polysaccharide that is widely used as a clinical anticoagulant. This glycosaminoglycan is prepared from animal tissues in metric ton quantities. Animal‐sourced heparin is also widely used in the preparation of low molecular weight heparins that are gaining in popularity as a result of their improved pharmacological properties. The recent contamination of pharmaceutical heparin together with concerns about increasing demand for this life saving drug and the fragility of the heparin supply chain has led the scientific community to consider other potential sources for heparin. This review examines progress toward the preparation of engineered heparins through chemical synthesis, chemoenzymatic synthesis, and metabolic engineering.

## 1 Introduction

### 1.1 Structure, activity, biosynthesis, and medical applications

**Figure 1:**
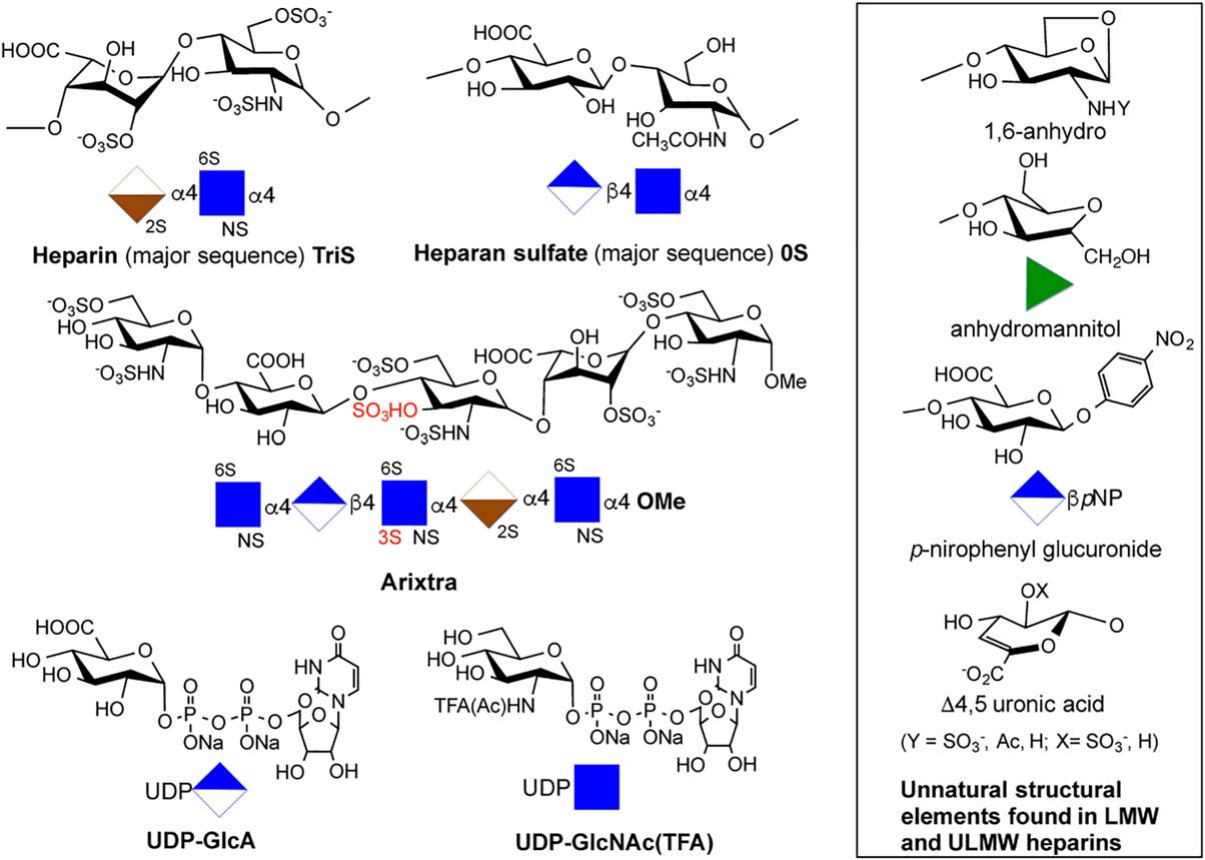
Chemical structures of GAG and intermediates used in their synthesis. The most common disaccharide structures comprising heparin and HS, the structure of Arixtra^®^, the structures of UDP sugars routinely used in GAG chemoenzymatic synthesis, and some unnatural saccharides found in LMW and ULMW heparins are shown

**Figure 2:**
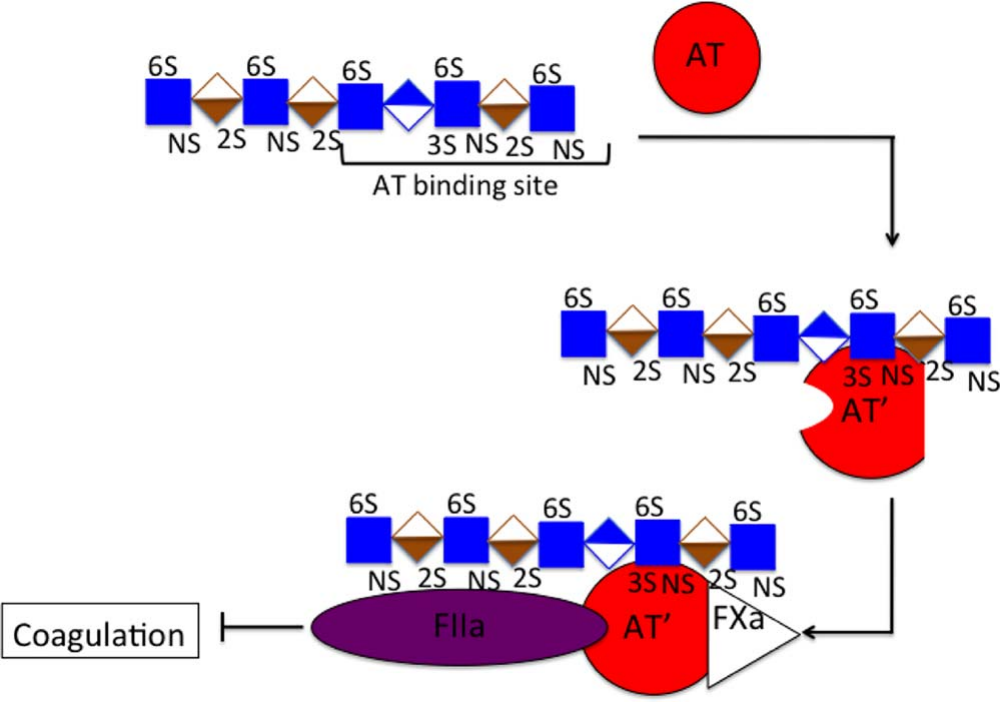
Anticoagulant activity of heparins. The heparin polysaccharide contains a pentasaccharide sequence, which is essential for the binding to AT. The 3‐*O*‐sulfo group at the central residue of this pentasaccharide unit is vital for this binding. Once bound, AT undergoes a conformational change to AT', which binds to either FXa or FIIa and blocks coagulation. FIIa must also bind to the heparin chain adjacent to the AT‐binding pentasaccharide to be effectively inhibited by AT'

Heparin is one of the most widely used anticoagulant drugs in medicine. A glycosaminoglycan (GAG), heparin is a linear polysaccharide comprised primarily (60‐80%) of a trisulfated (TriS) repeating disaccharide unit containing 2‐*O*‐sulfo‐α‐L‐iduronic acid (IdoA2S) 1,4‐linked to 6‐*O*‐sulfo‐*N*‐sulfo‐α‐D‐glucosamine (GlcNS6S) (Figure 1).^1^ In addition to this major repeating unit, heparin has approximately a dozen additional minor disaccharide units that result in a high level of structural, or sequence, heterogeneity. Particularly noteworthy is the 3‐*O*‐sulfo group, which is present at very low levels in heparin and is known to be critical for its anticoagulant activity (Figure 2).

In addition to its structural, or sequence, heterogeneity, heparin is a polydisperse biopolymer, and contains a mixture of polysaccharide chains of varying lengths, ranging from ∼16 to 160 saccharide units. The average molecular weight of heparin is approximately 20 kDa, corresponding to 30–40 disaccharide residues.^2^ Heparin can be chemically or enzymatically depolymerized to prepare low molecular weight (LMW) heparins having average molecular weights of 4–6 kDa, corresponding to 6–10 disaccharide residues^3^ (Figure 1). These LMW heparins have improved bioavailability and pharmacodynamics making them better than heparin for certain therapeutic applications.^4^ LMW heparins also show extensive structural and sequence variability and are polydisperse mixtures.^4^, ^5^, ^6^ Ultra‐low molecular weight (ULMW) heparins can similarly be prepared through a more extensive chemical or enzymatic depolymerization of heparin. These are polydisperse with average molecular weights of 2–3 kDa, corresponding to 3–5 disaccharide residues.^7^ A homogeneous ULMW heparin pharmaceutical, called Arixtra^®^ (fondaparinux), a pentasaccharide, (Figure 1) can be chemically synthesized.^8^

Heparin is only one member of the GAG family and is most closely related to heparan sulfate (HS), which contains all the disaccharides comprising heparin but in very different ratios. The major (generally >50%) structure is an unsulfated disaccharide (0S), β‐D‐glucuronic acid (GlcA) 1,4‐linked to *N*‐acetyl‐α‐D‐glucosamine (GlcNAc) (Figure 1). Both heparin and HS have domain structures consisting of high sulfate domains, called NS domains rich in TriS disaccharide and common in heparin, or low sulfate domains, called NA domains rich in 0S and common in HS. Other less closely related GAGs include chondroitin sulfate (CS), keratan sulfate (KS), and hyaluronan (HA).^9^

Heparin and HS are biosynthesized in the endoplasmic reticulum (ER) and Golgi through the same pathway.^10^ The heparin core protein, serglycin, is first biosynthesized in the rough ER.^11^ A tetrasaccharide linker (xylose‐galactose‐galactose‐GlcA, with xylose at the reducing end and GlcA at the non‐reducing end) is extended one sugar residue at a time from the non‐reducing end. There are multiple serine residues in the serglycin proteoglycan that contain heparin GAG chains. After construction of the linker region on the core protein, addition of α‐GlcNAc, to the non‐reducing end, by the enzyme α‐*N*‐acetylglucosaminyltransferase I, is followed by the action of a complex of two Golgi enzymes *EXT1* and *EXT2* that elongate the GAG chain by alternating addition of GlcA and GlcNAc residues.^12^ As chain elongation takes place the GAG backbone is modified through the action of a number of additional Golgi enzymes. First, the *N*‐acetyl groups are removed and replaced with *N*‐sulfo groups by *N*‐deacetylase/*N*‐sulfotransferase (NDST) enzymes The NDSTs are believed to be responsible for introducing the NS (repeating units having multiple *N*‐sulfo groups) and NA (repeating units having multiple *N*‐acetyl groups through the failure of their NDST removal) domains into heparin and HS chains.^12^ Next, uronosyl C5‐epimerase (C_5_‐epimerase) epimerizes some of the GlcA residues to IdoA residues.^13^ After epimerization, the GAG backbone is then variably sulfonated by a number of sulfotransferases that transfer a sulfo group from 3′‐phosphoadenosine 5′‐phosphosulfate (PAPS) onto specific hydroxyl groups within the chain. The 2‐OST first sulfonates the C2‐hydroxyl group of primarily IdoA residues, and to a lesser extent the GlcA residues.^14^ Next, the 6‐OSTs sulfonate the C6‐hydroxyl group of GlcNAc and GlcNS (and possibly GlcN) residues.^14^ Finally, the enzyme 3‐OST sulfonates the C3‐hydroxyl group of GlcNAc and GlcNS (and possibly GlcN) residues, which is required for the anticoagulant activity of heparin.^15^

Heparin's anticoagulant activity depends on a pentasaccharide sequence containing a central GlcN3S residue that binds to antithrombin III (AT) causing it to undergo a conformational change enhancing its ability to inhibit several coagulation cascade serine proteases, including thrombin (factor IIa) and factor Xa^1^ (Figures 1 and 2). Heparin, by definition has nearly equal anti‐factor Xa and anti‐factor IIa activities, while LMW heparins are selective anti‐Xa agents (anti‐factor Xa/anti‐factor IIa >1) and ULMW heparins are specific anti‐Xa agents with no anti‐factor IIa activity. In addition to its anticoagulant activity, heparin also exhibits anti‐inflammatory, anti‐atherosclerotic, anti‐infectious, anti‐ and pro‐proliferative, and anti‐metastatic properties.^16^ These activities are also mediated through heparin's interaction with proteins.^17^ Unlike heparin's anticoagulant activity, however, these other activities have not yet been therapeutically exploited.

### 1.2 Current methods used to produce heparin, LMW heparins and ULMW heparins

Pharmaceutical heparin is prepared from animal tissues that are rich in mast cells, in which heparin is biosynthesized as a proteoglycan attached to serglycin that is stored in mast cell granules.^18^ Animal tissues rich in mast cell heparin are generally tissues which have a high parasite burden, including, liver, lung, and intestine. It has been speculated that the main biological function of heparin is as an anti‐parasitic agent and also as a protection for the matrix by controlling mast cell proteolytic activity and storing histamine and other vasoactive amines found in the mast cell granules.^10^, ^19^ Currently, heparin is manufactured solely from porcine intestine, but in the past, bovine lung and bovine intestine have also been used as a source material for pharmaceutical heparin.^20^

There is approximately 30,000–50,000 U (∼300 mg per animal) of heparin in pig intestines collected at a slaughterhouse.^21^ In a typical process, salting of intestines is first used to preserve the tissues that are then solubilized using proteases. Heparin is captured either through precipitation with a hydrophobic quaternary ammonium salt or using an anion exchange resin. Heparin is resolubilized with saline and then repeatedly precipitated using alcohol to generate raw heparin, which is consolidated and shipped for purification at a pharmaceutical company operating under current good manufacturing practice (cGMP).^22^ Raw heparin is then processed into pharmaceutical grade heparin at the cGMP facility. Raw heparin is resolubilized, filtered to remove protein and bleached. Cation exchange resin is often employed to convert heparin to its sodium salt. Ethanol precipitation is used for nucleotide removal and residual salt is removed through membrane filtration and spray‐dried to afford pharmaceutical heparin.^21^

LMW heparins can be directly recovered from animal‐derived heparin by size exclusion chromatography, but such a process is unsuitable for large‐scale production. Instead, either chemical or enzymatic depolymerization of pharmaceutical heparin is used to prepare LMW heparins.^21^ Controlled, selective oxidation of heparin's uronic acid residues using reactive oxygen species often utilizes hydrogen peroxide‐based depolymerization. Deaminative cleavage with nitrous acid generates an anhydromannose residue at the reducing end of LMW heparin chains, which is subsequently reduced to an anhydromannitol residue (Figure 1). Heparin lyase, a bacterial enzyme, can be used for the controlled depolymerization of heparin through a β‐elimination cleavage mechanism. This enzymatic action can be mimicked using a chemical process in which the carboxyl group of uronic acid is first esterified, and base treatment leads to selective β‐eliminative cleavage. The LMW heparins generated using both enzymatic and chemical β‐elimination have a characteristic unsaturated Δ4,5 uronic acid residue at their non‐reducing end (Figure 1). Chemical β‐elimination also produces an unnatural 1,6‐anhydro residue at the reducing end of some of the LMW heparin chains (Figure 1).

Polydisperse ULMW heparins can be prepared through more extensive depolymerization of long chained heparin.^7^ Arixtra^®^ (fondaparinux), a homogeneous ULMW heparin, was introduced as a new anticoagulant drug in 2001. Its multi‐step chemical synthesis leads to high production costs, making it far costlier than heparin or LMW heparins.^8^ The expiration of patent protecting Arixtra^®^ has paved the way for the development of generic versions at reduced costs.

### 1.3 Evaluating the anticoagulant activity of heparin products

The anticoagulant activities of heparin, LMW heparins and ULMW heparins are different primarily as the result of their chain length. All anticoagulant heparin products contain an AT pentasaccharide binding‐site having a critical central GlcN3S residue. While there are a number of structural variants of the AT pentasaccharide binding‐site, a detailed study on the structure‐activity relationship of these variants have not been reported.^23^, ^24^ While factor Xa can be inhibited by the binary AT‐pentasaccharide complex, factor IIa (thrombin) requires the assembly of a ternary factor IIa‐AT‐heparin oligosaccharide (14–16 saccharide residues) complex^16^ (Figure 2). Anti‐factor Xa and anti‐factor IIa activities were originally determined in plasma‐based coagulation assays containing AT and enriched in these serine proteases. In this assay fibrinogen is converted by a serine protease, thrombin, into a fibrin clot and the time to clot formation is determined. The modern means for determining anti‐factor Xa and anti‐factor IIa activities are performed in the absence of the plasma protein fibrinogen and instead rely on the amidolytic cleavage of *p*‐nitroaniline‐labeled synthetic peptide substrate to release the *p*‐nitroaniline chromophore. The anti‐factor Xa and anti‐factor IIa activities are determined by kinetic or endpoint assays from a standard curve obtained by incubating either pure factor Xa or factor IIa and *p*‐nitroaniline‐labeled synthetic peptide substrate with an excess of pure AT and a limiting amount of heparin product.

## 2 Chemoenzymatic synthesis of heparin

### 2.1 Polysaccharide backbone synthesis

#### 2.1.1 Overview

The chemoenzymatic synthesis of heparin generally involves a two‐part process, the preparation of the polysaccharide and subsequent chemical and/or enzymatic modification of that backbone to remove most of the *N*‐acetyl groups and add *N*‐sulfo groups to the resulting GlcN residues, epimerize most of the GlcA residues to IdoA residues and introduce *O*‐sulfo groups to the 2‐position of IdoA residues and 6‐ and 3‐positions of GlcN residues. Several approaches have been undertaken to accomplish these steps.

#### 2.1.2 Fermentation for the preparation of polysaccharide backbone

**Figure 3:**
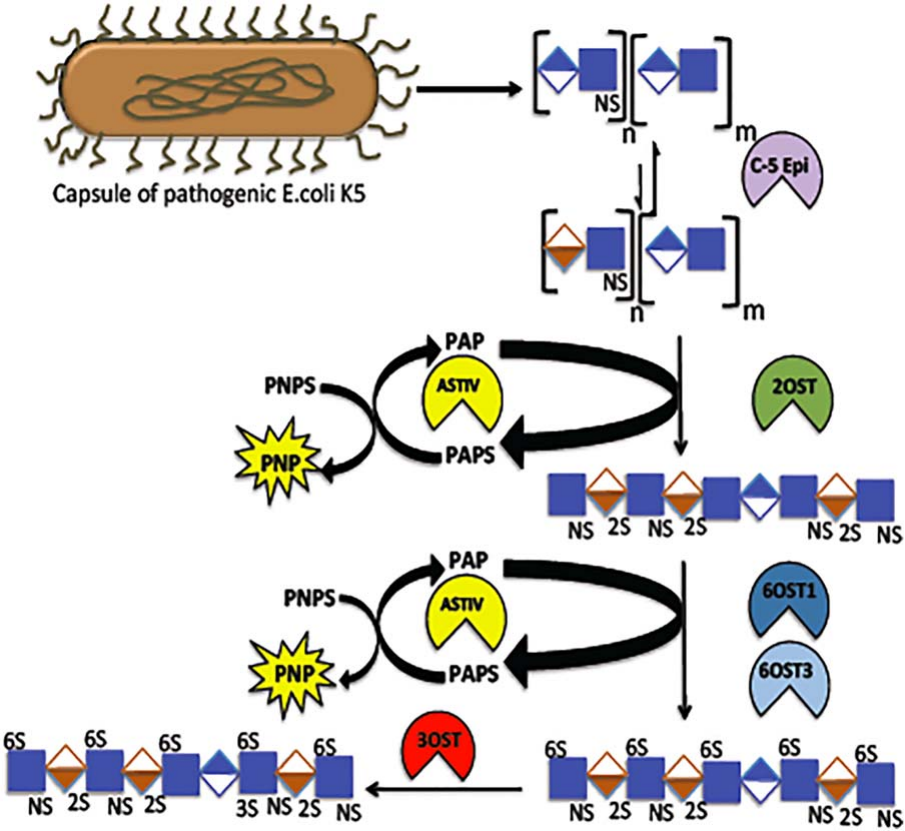
A schematic showing the chemoenzymatic synthesis of heparin from *N*‐sulfoheparosan. The backbone is first produced from the capsule of *E. coli* K5. This is then purified and *N*‐sulfonated chemically. The resulting polysaccharide *N*‐sulfoheparosan is then treated with a series of enzymes beginning with C5‐Epi, 2‐OST, 6OST1, 6OST3, and 3OST1 to produce anticoagulant heparin. The chemoenzymatic scheme for the synthesis of heparin involves the biosynthesis of the substrate by utilizing the capsule of pathogenic *E. coli* K5. As PAPS gets utilized quickly, PNPS acts as a sacrificial sulfur donor for the recycling of PAPS.^47^ This reaction is catalyzed by arylsulfotransferase IV

The *Escherichia coli* K5 capsular polysaccharide (CPS)^25^ is heparosan of average molecular weight 75–150 kDa^26^ with a disaccharide repeating structure GlcA 1,4‐linked to GlcNAc and, thus, can be utilized as the polysaccharide backbone for heparin synthesis after reduction of its molecular weight (Figure 3). The in vivo bacterial biosynthesis of heparosan is initiated on a 2‐keto‐3‐deoxyoctulosonic acid glycolipid acceptor.^27^, ^28^ Bacterial heparosan is elongated with repeating units of GlcNAc and GlcA through the sequential action of polysaccharide synthases KfiA and KfiC. This polysaccharide can be released through the action of an enzyme called K5 lyase. In cases where the gene encoding this enzyme is integrated into the *E. coli* K5 genome through a bacteriophage infection, the expression of K5 lyase needs to be monitored to control the production of the backbone. The lyase acts on the polysaccharide by β‐elimination thereby causing a release and the shortening of the heparosan chain. Alternatively, heparosan molecular weight can be reduced by controlled chemical cleavage.^29^

Since the heparosan polysaccharide backbone prepared through fermentation consists of a uniform structure, with a single 0S block, it must be selectively modified to introduce domains or motifs that can be more fully elaborated. The selective modification of the heparosan backbone often begins with chemical *N*‐deacetylation using strong base and *N*‐sulfonation using Et_3_N‐SO_3_ to produce an *N*‐sulfo‐*N*‐acetyl heparosan, a suitable substrate for enzymatic conversion to heparin^30^ (Figure 3). The resulting chain can be prepared to contain different amounts of NA and NS based on hydrolysis conditions but the position and clustering of these domains is not possible using such methods. Enzymatic treatment of bacterially produced heparosan with NDST‐1 or NDST‐2 can similarly afford *N*‐sulfo‐*N*‐acetyl heparosan with different domains arising from the different specificities of the NDST isoforms.^31^

#### 2.1.3 Block synthesis

**Figure 4:**
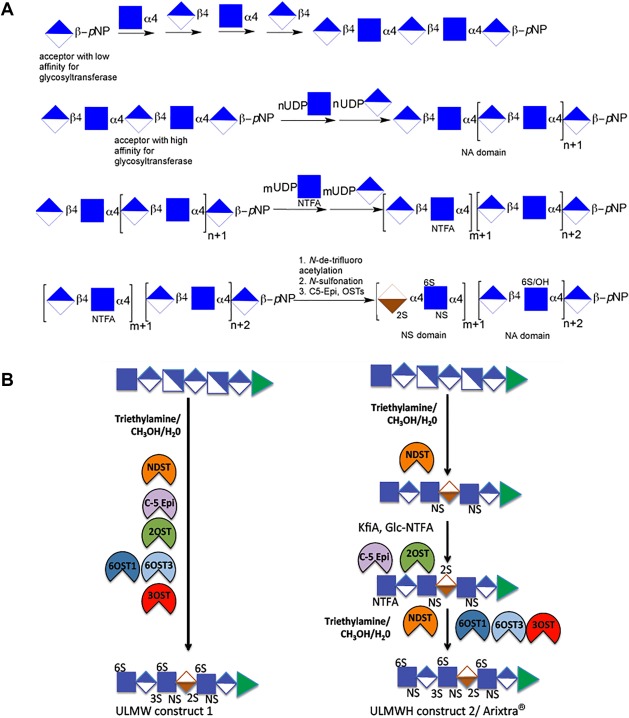
Chemoenzymatic synthesis of heparin and heparin oligosaccharides. (A) Block synthesis—The core polysaccharide is assembled by the initial binding of a bi‐catalytic glycosyltransferase to an acceptor (rate‐determining step). In the first set of reactions a poor acceptor is first iteratively elongated to prepare a good acceptor that binds tightly to the bi‐catalytic glycosyltransferase. This good acceptor is then elongated through the rapid kinetically alternating addition of natural (UDP‐GlcNAc/UDP‐GlcA) or unnatural (UDP‐GlcNTFA) UDP‐sugar donors to the acceptor creating a long chained core polysaccharide. The reaction terminates after the donor pool is depleted and the product chain length can be well controlled by adjusting the stoichiometry of the moles of acceptor to moles of donor.^32^ Finally, the *N*‐TFA groups are removed and replaced with *N*‐sulfo groups to obtain a two‐domain (NA and NS) block polysaccharide. (B) Iterative synthesis—ULMW heparin synthesis utilizes a combination of both concepts of glycotransferase catalyzed synthesis of the core oligosaccharide (see top line in Figure 4A) followed by controlled chemoenzymatic modification of this core oligosaccharide using the same enzymes relied upon for preparing bioengineered heparin. The synthesis of two homogenous ULMWHs having structures and activities similar to the commercially available pharmaceutical ULMWH, Arixtra^®^, are shown.^35^ Both these ULMW constructs are built on the disaccharide acceptor 2,5 anhydromannitol (1,4) GlcA that was prepared from heparosan. See Figure 1 for the structures of 2,5 anhydromannitol and Arixtra^®^. Similar syntheses have been carried out using *p*‐NP‐GlcA acceptor (as in Figure 4A, see Figure 1 for the structure)^4^, ^36^

The backbone polysaccharide for heparin can be enzymatically synthesized in vitro using bacterial polysaccharide synthases (Figure 4). In such syntheses, uridine diphosphate (UDP)‐GlcNAc and UDP‐GlcA (Figure 1) can be sequentially transferred onto a monosaccharide or an oligosaccharide acceptor.^32^ Kinetic control of chain extension is possible, in which synthase is bound to acceptor (slow step) and then UDP‐GlcNAc and UDP‐GlcA are added and chain extension takes place (fast step) (Figure 4A). Product chains of nearly uniform and predictable size can be prepared by controlling the amounts of the UDP sugars and making it the limiting reagent.^32^ Synthases, or glycosyl transferases, have been prepared from microorganisms including, *Pasteurella multocida*, PmHS1, PmHS2, and *E. coli*, KifA and KifC, using recombinant DNA technology.^33^ In addition to adding their natural substrates, UDP‐GlcNAc and UDP‐GlcA, these enzymes can also accept unnatural substrates, such as UDP‐*N*‐trifluoroacetylglucosamine (UDP‐GlcNTFA) (Figure 1).^34^, ^35^ Unlike GlcNAc, which is *N*‐deacetylated only on treatment with a strong base like sodium hydroxide, GlcNTFA is readily *N*‐detrifluoracetylated with a mild base like triethylamine.^34^, ^35^ The resulting GlcN residues can then be chemically sulfonated using Et_3_N‐SO_3_ or enzymatically sulfonated using *N*‐sulfotransferase (NST prepared by removal of the *N*‐deacetylase domain from and NDST).^21^ Thus, the incorporation of GlcNTFA offers a method of introducing domain structures into a heparosan chain that mimics the biosynthetic introduction of such domains through the controlled action of NDSTs.

Such an approach may, for example, begin with transferring UDP‐GlcNAc and UDP‐GlcA to an acceptor such as *p*‐nitrophenyl (*p*NP)‐GlcA (Figure 4A). After extending the chain to obtain a high affinity acceptor, a longer chain (i.e., 5 kDa or ∼12 disaccharide units) consisting of an NA domain is prepared under kinetic and stoichiometric control (limiting amounts of UDP‐GlcNAc to ensure it was all depleted). Next an NS domain precursor is similarly prepared by chain extension with UDP‐GlcNTFA and UDP‐GlcA. The resulting chain can then be treated with triethylamine to selectively remove TFA groups, followed by Et_3_N‐SO_3_ to afford a two‐domain (NA and NS) 10 kDa chain of (reducing end) *p*NP‐[GlcA‐GlcNAc]_11‐13_[GlcA‐GlcNS]_11‐13_GlcA (non‐reducing end).^34^ This in vitro chain synthesis is rather expensive due to the cost of the UDP‐sugars, which themselves require chemoenzymatic synthesis. An advantage of this method is that it can be performed under kinetic/stoichiometric control and affords nearly monodisperse products containing domain structures similar to those present in heparin and HS.

#### 2.1.4 Iterative synthesis

Stepwise or iterative in vitro synthesis of the heparosan backbone is also possible (Figure 4A, top set of reactions). This method can be performed by the addition of a single UDP‐sugar at a time onto a monosaccharide or an oligosaccharide acceptor resulting in the controlled elongation of the backbone to prepare a specific homogenous target structure.^4^, ^36^ For example, a homogeneous chain of the structure *p*NP‐GlcA[GlcNAc‐GlcA‐GlcNS‐GlcA]_4_ might be prepared on a *p*NP‐GlcA acceptor through iterative alternating addition UDP‐GlcNAc, UDP‐GlcA, UDP‐GlcNTFA, and UDP‐GlcA repeated four times. This method is both time consuming (typically requiring purification of intermediates formed after each sugar addition) and also expensive, but it represents the best way to synthesize a library of defined homogeneous chains. Moreover, it allows for the exact positional control of NS and NA domains. This method is capable of producing small, structurally defined heparin polymers of sizes of <5 kDa.

### 2.2 Elaboration of the backbone polysaccharide

#### 2.2.1 Overview

The polysaccharide backbones prepared through fermentation/chemical modification, in vitro block synthesis and in vitro iterative synthesis can each be elaborated enzymatically to prepare heterogeneous heparin chains resembling a pharmaceutical heparin called bioengineered heparin (Figure 3),^37^ nearly homogenous heparin,^38^ HS containing well‐positioned block structures,^34^ or shorter homogeneous heparins or HS chains.^4^, ^36^ Once the polysaccharide backbone has been prepared by one of these methods, the next challenge is to epimerize selected GlcA residues to IdoA residues through the action of C‐5 epimerase (C5‐Epi)^39^ and to introduce *O*‐sulfo groups to the 2‐position of the uronic acid residues using 2‐*O*‐sulfotransferase (2OST)^39^ and PAPS, most prominently to prepare IdoA2S. In addition, the 6‐ and 3‐positions of the GlcN residues are sulfonated using 6‐*O*‐sulfotransferases (6OSTs)^40^ and 3‐*O*‐sulfotransferases (3OSTs) and PAPS, respectively, most prominently to obtain GlcNS6S and GlcNAc6S residues. Minor residues, including GlcNX6S3S and GlcNAc6X3S residues (where X = S or OH) and GlcA2S occasionally may need to be prepared to obtain rare but potentially biologically and pharmacologically important sequences. The multiple isoforms of the 6OSTs and 3OSTs have different and still not fully understood specificities.^15^, ^41^ Thus, the controlled application of these enzymes to prepare desired target structures remains challenging. Typically, the stepwise conversion of an *N*‐sulfo‐*N*‐acetyl heparosan into a heparin product requires the isolation, purification and characterization of all the intermediates generated, including that from the C5‐Epi/2OST and 6OST steps as well as the final product generated after the 3OST step (Figure 3).

#### 2.2.2 Enzymes involved in chain modification—C5 epimerase and OSTs

The preparation of heparin from an *N*‐sulfo‐*N*‐acetyl heparosan backbone initially involves the action of glucuronosyl C5‐Epi.^13^ This enzyme catalyzes the reversible and irreversible conversion of GlcA to IdoA depending on the context of the site at which C5‐epi acts.^42^ The presence of an adjacent GlcNS residue is required for GlcA both reversible and irreversible C5‐epimerization and upstream GlcNAc residue is required for irreversible C5‐epimerization. After an IdoA residue is formed within the polysaccharide backbone it can be locked in place through the action of 2OST to form IdoA2S, which is not a substrate for C5‐Epi, thus preventing its conversion back to a GlcA residue^43^ GlcA is a poor substrate for 2OST so that even extensive treatment of *N*‐sulfo‐*N*‐acetyl heparosan backbone with 2OST in the presence of PAPS gives only a small amount of GlcA2S containing product.^44^ Furthermore, if an *N*‐sulfo‐*N*‐acetyl heparosan backbone is first treated with 6OST‐1 or 6OST‐3 introducing GlcNS6S and GlcNAc6S residues, then the resulting intermediate can no longer undergo C5‐epimerization and 2‐*O*‐sulfation.^44^

After the formation of an IdoA2S‐containing *N*‐sulfo‐*N*‐acetyl heparosan backbone the 6OSTs can then act to afford the major TriS disaccharide‐repeating unit, IdoA2S‐ GlcNS6S, making up most of the heparin polysaccharide. The context‐dependent subspecificities of three 6OST isoforms, 6OST‐1, ‐2, and ‐3, are not well understood but all can introduce, with differing efficiency, 6‐*O*‐sulfo groups into both GlcNAc and GlcNS residues.^44^ The final step in the preparation of pharmaceutical heparin having anticoagulant activity requires the action of the 3OST‐1 isoform at a GlcNS6X or GlcNAc6X residue two residues upstream of a GlcA residue to afford an AT pentasaccharide binding site. The specificity of the other six 3OST isoforms (3OST2‐7) are less well studied.^42^, ^45^, ^46^

#### 2.2.3 Cofactor regeneration and synthesis of UDP sugars

The sulfotransferases all require PAPS that acts as a sulfo donor. PAPS can be enzymatically synthesized by using two moles of adenosine triphosphate (ATP) in a buffer containing sulfites in the presence of adenosine 5′‐phosphosulfate (APS) kinase and ATP sulfurylase, and inorganic phosphates and phosphoenolpyruvates.^47^ When working at scales that require more than tens of milligrams of PAPS, it is most cost effective to use a catalytic quantity of PAPS and to regenerate it enzymatically using an inexpensive sacrificial sulfo donor *p*‐nitrophenylsulfate (PNPS) (Figure 3). PAPS regeneration utilizes a recycle system involving PNPS and aryl sulfotransferase (AST‐IV) to convert the product formed in the sulfotransferase reaction, 3′‐phosphoadenosine 5′‐phosphate (PAP), back into PAPS and *p*‐nitrophenol (PNP), a yellow colored product that can be conveniently detected at 400 nm. This provides for both an efficient use of the expensive PAPS cofactor as well as a convenient assay for OST activity.^44^

The UDP‐sugars (Figure 1), UDP‐GlcNAc, UDP‐GlcA, UDP‐GlcNTFA, and UDP‐GlcA, required for the in vitro enzymatic synthesis of heparosan polysaccharide backbone can be chemoenzymatically synthesized.^35^ Briefly, these UDP sugars are prepared by GTases or GAG synthases. This process is the enzymatic synthesis using recombinant technology. These UDP sugars and their analogs can also be synthesized chemically but this is a tedious process with poor yields.^35^

### 2.3 Major advances in the field of bioengineered heparin

#### 2.3.1 One‐pot synthesis of heparin

While initial studies focused on the stepwise conversion of the *N*‐sulfo‐*N*‐acetyl heparosan backbone intermediate to heparin, a one‐pot synthesis has also been evaluated. A one‐pot synthesis offers a distinct advantage in that it allows the production of heparin without the isolation, purification, and characterization of all the intermediates and requires only the purification of the final product. The phased addition of enzymes allows the *N*‐sulfo‐*N*‐acetyl heparosan substrate to be correctly converted into heparin product.^48^ While this approach gives an anticoagulant heparin product, additional optimization will be required to ensure its equivalency to pharmaceutical heparin. Moreover, when working with homogeneous *N*‐sulfo‐*N*‐acetyl heparosan substrate a one‐pot approach would undoubtedly lead to product mixtures.

#### 2.3.2 Immobilized enzymes for enhanced production of bioengineered heparin

The chemoenzymatic synthesis of heparin has recently been further optimized by using immobilized biosynthetic enzymes. Each of the biosynthetic enzymes as well as AST‐IV have been successfully immobilized with retention of activity.^49^ Immobilized enzymes typically show enhanced stability often allowing their recovery and reuse. Furthermore, intermediate and product purification can be simplified as enzymes can be removed by filtration. Spent cofactors, such as PAP and PNP can also be removed from the polysaccharide product through dialysis allowing for a clean and economical synthesis.

#### 2.3.3 ULMW heparins and LMW heparins having defined, homogeneous structures

Utilizing this chemoenzymatic scheme, a range of ULMW heparins and LMW heparins have also been synthesized in high yields with homogenous structures (Figure 4B).^4^, ^36^ In these syntheses, the core polysaccharides were chemoenzymatically synthesized in vitro using an acceptor and UDP sugars in an iterative process. The targets of these contained the AT binding site in the case of both ULMW heparin and LMW heparin targets. The ULMW heparin targets behaved analogously in both in vitro and in vivo studies to Arixtra^®^ and could be synthesized in far fewer steps and in greater yield than the commercial ULMW heparin product.^36^ The LMW heparin targets also contain a TriS domain adjacent the AT binding site to improve their biological properties. The inclusion of this domain allowed for the complete reversibility of these chemoenzymatically synthesized LMW heparins with protamine, a heparin antidote, which is completely ineffective in the neutralization of Arixtra^®^ and only partially effective in the neutralization of commercial LMW heparin products.^4^ Moreover, unlike commercial LMW heparins or ULMW heparins, these chemoenzymatically synthesized LMW heparins could be cleared through the liver, possibly facilitating their use in renal compromised patients.

## 3 Metabolic engineering

### 3.1 Overview and advantages of metabolic engineering

**Figure 5:**
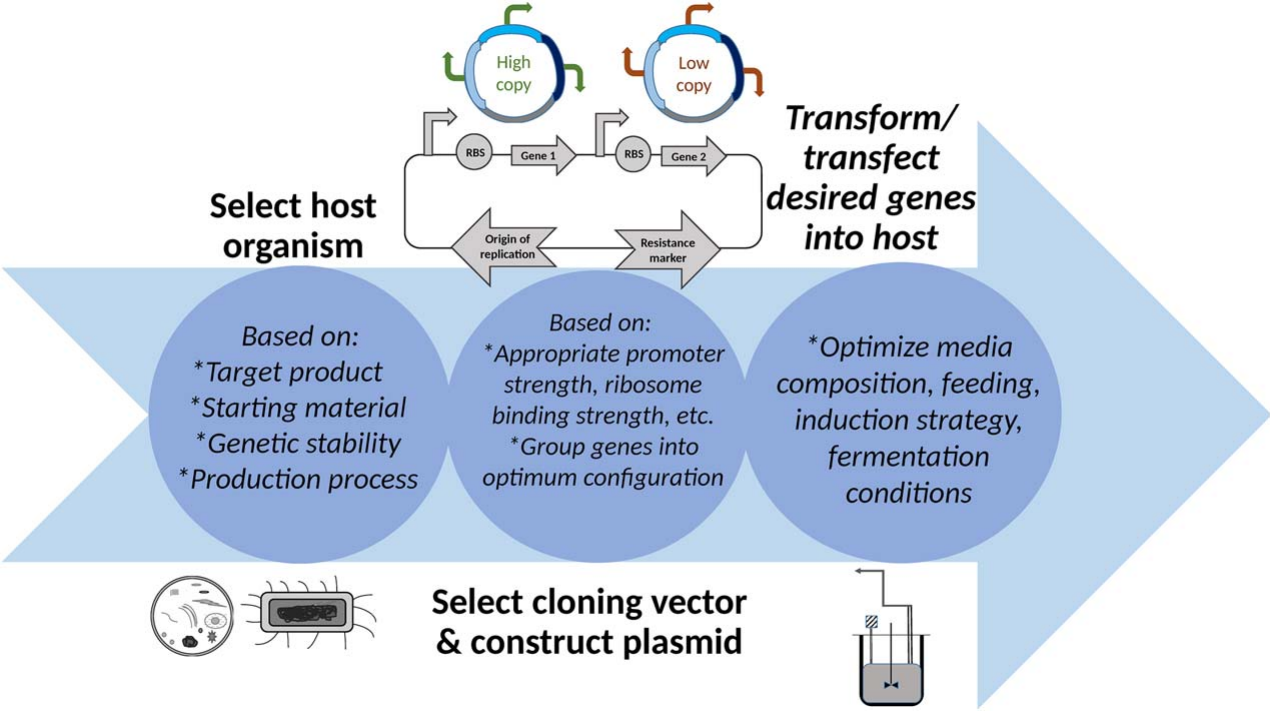
Overview of the metabolic engineering process is shown

The process of metabolic engineering is targeted toward overexpressing specific gene pathways that result in the production of a desired product, while suppressing competing pathway.^50^, ^51^ Generally, this is achieved through the transfer of product‐specific enzymes or complete metabolic pathways from an often inflexible host organism into a more easily manipulated and readily available engineered microorganism, thereby facilitating the efficient manufacture of various products, including valuable small molecules and nutraceuticals.^52‐54^ Figure 5 illustrates a general overview of the metabolic engineering process. The final yield of the target product can be augmented by metabolic pathway balancing in the chosen host organism using a combination of traditional approaches like promoter engineering, as well as more contemporary methods like dynamic balancing and compartmentalization.^55^, ^56^ Synthetic biology techniques are also becoming increasingly valuable tools for pathway optimization and metabolic engineering applications.

Increasing safety concerns have led to a movement away from traditional animal‐sourced methods of GAG production, due to the associated drawbacks of high interspecies viral contamination risk and inconsistent product quality and activity.^22^ Metabolic engineering plays an important role in the development of strains that use recombinant technologies to synthesize polysaccharides such as heparin, HA, and CS.^57^ The sulfated GAGs, heparin and CS, have more intricate chemical compositions than the simple repeating disaccharide unit of HA. Much work has been focused on developing various mammalian and bacterial strains that can manufacture non‐sulfated heparin‐precursor, heparosan, and the backbone of the CS‐precursor, chondroitin, by fermentation. These precursor molecules can then be modified using biosynthetic pathway enzymes and other required elements to produce the desired final product.^58^ Table 1 summarizes the results to date on the production of GAGs and their polysaccharide precursors through the use of metabolic engineering.

**Table 1:** List of relevant polysaccharides obtained through metabolic engineering techniques and their yields

**CPS or GAG product**	**Microorganism or cell line**	**Maximum reported yield**
Chondroitin	*E. coli* K4 → *E. coli* BL21^59^	2.4 g/L
	*B. subtilis* 60	5.22 g/L
Heparosan	*E. coli* BL21^61^	1.88 g/L
	*E. coli* K5^28^	15 g/L
	*B. subtilis* 60	5.82 g/L
	*E. coli* K‐12^62^	1 g/L
HS/heparin	CHO‐S cells (culture media)^63^	173.2 μg/(5 × 10^7^ cells of cell line)
CS/DS	CHO‐S cells (cell pellet)^63^	2.2 μg/(5 × 10^7^ cells of cell line)

Further work by Baik and coworkers was able to achieve a maximum final GAG concentration of ∼90 μg/ml with bioprocess optimization techniques; however the product composition varied from that of pharmaceutical heparin.^64^

### 3.2 Heparin and heparin‐like polysaccharides—CPSs

Heparin and HS share a similar biosynthesis pathway and both possess a common preliminary GAG chain, which can undergo further modification to differentiate into either highly *N*‐sulfo, *O*‐sulfo, IdoA‐rich heparin chains, or *O*‐sulfo poor and GlcNAc, GlcA‐rich HS chains.^64^ While the heparin polysaccharide is primarily found intracellularly within mast cell granules, HS is located extracellularly and in the cell membrane, and their precursor molecule, heparosan is only present as an intermediate in the Golgi.^10^ Because heparin is produced as a proteoglycan in eukaryotic mast cells Golgi, it is possible to biosynthesize it in eukaryotic systems like insect cells, Chinese hamster ovary (CHO) cells, and potentially yeast, but it is currently not technologically feasible to genetically engineer it in bacteria.^3^, ^21^ However, heparosan, like chondroitin and hyaluronic acid, is a CPS that can be produced by bacterial cells. These bacterial capsules serve as the principal protection against intrinsic host defense within the cell surface.^59^

### 3.3 Biosynthesis within eukaryotic systems/mammalian cells

#### 3.3.1 Yeast cells and heparin or HS biosynthesis

Although yeast strains are capable of producing vital glycosylation patterns in mammals and can be used as recombinant protein expression systems, yeast cells do not produce heparin or HS. Biosynthesis of heparin or HS in yeast cells would be very difficult since it would entail the high‐level expression of core proteins along with the measured expression of all the enzymes present in the heparin/HS biosynthetic pathway.^21^

#### 3.3.2 Cultured murine cell lines—murine mastocytoma cell line for heparin production

Murine mastocytoma (MST) cell lines can shed some light on the biosynthesis pathway that produces heparin in mast cells, since they naturally produce a highly sulfated polysaccharide that resembles heparin.^65^ MST cells express the genes *Ext1, Ndst2, Hs2st1,* and *Hs6st1*, which are responsible for the production of highly sulfated HP chains, but do not possess the heparan sulfate‐glucosamine 3‐sulfotransferase 1 (*Hs3st1*) gene that is present in pharmaceutical heparin and required for anticoagulant activity.^66^ An MST clone into which the murine *Hs3st1* gene was transfected (MST‐10H cell line) was found to show substantially more anticoagulant activity than MST cells without the *Hs3st1* gene. The anticoagulant activity of heparin is governed by the presence of AT binding sites that contain a 3‐*O*‐sulfo group, which was shown by structural analysis to be present in the MST‐10H clone but not the MST cell line. This confirms that the *Hs3st1* gene is responsible for the observed spike in anticoagulant activity in the heparin product of the clone.^65^ Stable cell lines that can produce heparin have also been derived from the Furth MST, with the majority of the GAG product being stored in cytoplasmic granules.^67^

#### 3.3.3 Metabolic engineering of CHO cells

##### 3.3.3.1 Heparin production through heparan sulfate biosynthetic pathway

CHO cells are mammalian host cells, which are frequently used for the production of non‐native proteins. Their use in therapeutic glycoproteins is established and they are relatively safe from biological contamination, like viruses. The suitability of CHO cells for GAG production stems from their ease of culture and the fact that they are able to express many glycosylation enzymes.^68^ The ability of these cells to produce HS, a less sulfated polysaccharide that shares a comparable biosynthesis pathway and disaccharide structure with heparin, introduces the possibility of metabolically engineering CHO‐S cells to produce heparin through the development of stable cell lines that express the required enzymes.^64^, ^67^

Dual expressing cell lines (clones) were obtained by consecutively transfecting CHO‐S cells with human *N*‐deacetylase/*N*‐sulfotransferase (*NDST2*) and mouse heparan sulfate glucosamine 3‐*O*‐sulfotransferase 1 (*Hs3st1*) genes, two genes that are not expressed natively in CHO‐S cells and code for necessary biosynthesis enzymes. Compared to the parental CHO‐S cell line, the engineered clones showed increases in anticoagulant activity of ∼10‐fold from the cell pellets and ∼100‐fold from the culture medium, although the *NDST2* was too active and the overall anticoagulation activity was still lower than that of pharmaceutical heparin. This dearth of activity was possibly due to a *Hs3st1* mistargeting issue, resolved by making the *Hs3st1* Golgi‐targeted, leading to an increase in 3OST‐1 expression.^69^ This targeting of 3OST‐1 expression to the Golgi may also lead to the upregulation of native OSTs such as 2‐OST and 6‐OS, resulting in further improvements in anticoagulant activity.^70^

The amount of GAG released into the culture media was greater than the amount extracted from the cell pellets, indicating that bioengineered GAG chains are directed toward the cell exterior by utilizing core proteins. Thus, overexpression of the core proteins may be required to increase movement of the bioengineered HS/heparin and increase product yield.^64^ Not only was metabolic engineering able to increase the metabolic flux through the pathway, but product purification was also simplified since cell lysis would no longer be required for product recovery. Further pathway and enzyme expression balancing is needed to obtain a bioengineered HS product that more closely resembles pharmaceutical heparin. The possibility for achieving greater control of expression levels exists through use of an inducible system that permits concurrent optimization of *NDST2* and *Hs3st1* expression.^64^

##### 3.3.3.2 Bioprocess optimization with metabolic engineering enhancements

Further increases in yield and activity of bioengineered heparin can be achieved by metabolic engineering fine‐tuning coupled with bioprocess optimization. Work done by Baik and coworkers demonstrated how changes in fermentation conditions, feeding strategy, and media composition can significantly affect product titers.^63^ For example, when cysteine, a source of sulfur, is added to shake flask experiments with engineered CHO‐S cell lines, the cultures enriched with cysteine were found to have better anticoagulant activity than those without cysteine. Additionally, allowances have to be made for variances in the metabolic behavior of parental CHO cells and engineered CHO cell lines, which often lead to differences in nutrient uptake and metabolite formation. Despite the substantial increases in yield and productivity attained through process optimization, the composition of the bioengineered product still varied from that of pharmaceutical heparin, indicating that room for improvement still exists on the metabolic engineering front.^63^

### 3.4 Metabolic engineering of prokaryotic cells

#### 3.4.1 *E. coli* K4 strain genes for chondroitin and chondroitin‐like CPS production as a model system for metabolically engineering heparin

##### 3.4.1.1 *E. coli* K4 strain genes for chondroitin and chondroitin‐like CPS production

The CPS of *E. coli* K4 has a disaccharide repeat unit that is equivalent to fructosylated chondroitin, presenting an avenue for chondroitin production by microbial fermentation.^70^ The use of this microbial system as a CS source is a safer and cheaper alternative to animal‐sourced CS, with a simple *kfoE* gene knockout resulting in the unfructosylated chondroitin product.^59^, ^71^ The ePathBrick system, a multigene pathway manipulation tool for efficient pathway optimization, was used to construct the *E. coli* K4 biosynthetic pathway. The system's highest copy number vector, pETM6, was used in a pseudo‐operon configuration, where genes are individually controlled by different promoters but all share a single terminator for the mRNA transcripts.^72^ K4 CPS is a member of the group 2 K antigens, where region II of the gene cluster contains the genes encoding CPS synthesis and assembly enzymes.^59^, ^73^ Maximum yield was obtained when the three essential chondroitin biosynthesis genes were arranged in the order *kfoC, kfoA, kfoF* in a pETM6_PCAF construct, and expressed in the non‐pathogenic *E. coli* BL21 Star (DE3) strain. Chondroitin production reached maximum levels of 2.4 g/L in fed batch fermentation and 213 mg/L in shake flasks. Since each transcript finishes at a common terminator positioned downstream of the last gene, it can be deduced that the extent to which each gene is transcribed decreases in the order *kfoF, kfoA, kfoC*, with *kfoC* being the least transcribed gene.^59^

##### 3.4.1.2 rfaH overexpression

The three genes of functional importance in region 2 of the group II K antigen gene cluster are *kfoA, kfoF,* and *kfoC*. Respectively, these genes code for a UDP‐glucose‐4‐epimerase, a UDP‐glucose dehydrogenase involved in nicotinamide adenine dinucleotide (NAD^+^) and UDP‐glucose redox reactions, and chondroitin polymerase which catalyzes both chondroitin polymerization and glycosylation.^59^ In conjunction with metabolic engineering, transcriptional management of the K4 CPS gene cluster by transcriptional regulators such as *SlyA* and the antitermination transcriptional factor *rfaH* can lead to improved production of K4CPS in *E. coli* K4, particularly during the stable phase of growth. A similar strategy can also be employed to enhance the production of other group II polysaccharides, such as heparosan, a precursor to heparin.^71^ The transcriptional activator *rfaH* is responsible for the antitermination process in capsule expression, and its homologous overexpression in *E. coli* K4 leads to a substantial increase in the yield of CPS, through its impact on the intracellular concentration of UDP‐sugar precursors.^28^

##### 3.4.1.3 ePathBrick method for optimization of metabolic pathways

Synthetic biology techniques are becoming increasingly valuable tools for application to metabolic engineering and pathway optimization. One such tool is the ePathBricks system, a modular platform for DNA assembly that employs isocaudomer restriction enzyme pairs to construct vectors that allow expression and cloning of full pathways in three operon configurations.^74^, ^75^ At the level of the individual genes, control elements including promoters, operators, ribosome binding site, and terminators, permit direct pathway manipulation and greater control of strain design, consequently allowing the full capacity of cell metabolism to be taken advantage of. The output from a particular multigene biosynthesis pathway depends more heavily on its fundamental genetic arrangement and the order of the pathway genes, than on the availability of required precursors. The pseudo‐operon configuration (multiple promoters and one terminator) was found to yield the largest amount of product, followed by the monocistronic form (multiple promoters and terminators), and then operon (one promoter and one terminator for all genes) with the lowest product yield.^72^ Moreover, different gene orders in a given configuration, like pseudo operon, can result in varying expression levels of each gene, as illustrated in the pETM6_PCAF construct used for K4 CPS biosynthesis.^59^

#### 3.4.2 *E. coli* K5 strain for heparosan production

Heparosan production is commonly viewed as the first step in making bioengineered heparin in eukaryotes. This precursor polysaccharide is made up of a repeating disaccharide unit of GlcA 1,4‐glycosidically linked to GlcNAc. Heparosan is a CPS biosynthesis product of both *E. coli* K5 and *Pasteurella multicida,* but *E. coli* K5 is the preferred host organism since its heparosan product is closer in size to heparin. The region 2 genes of the K5 gene cluster are responsible for the biosynthesis of capsular heparosan polysaccharide by *E. coli* K5.^73^ Through bioprocess optimization, the heparosan yield from *E. coli* K5 fermentation was increased to 15 g/L, improving on a patent by Viskov and coworkers.^76^ Metabolic engineering of the strain can lead to further increases in product yield. The region 2 *kfiA* and *kfiC* genes are responsible for lengthening the heparosan chain at its non‐reducing end, so it is plausible that the quantities of these two glycotransferases and their activities would constrain the manufacture of heparosan, pointing to a need for their overexpression to be carefully balanced. Enhanced heparosan release from the cell surface into the medium can also boost yields, and can be most feasibly achieved through expression of a lyase gene integrated into the *E. coli* K5 DNA. Since lyases also lead to a decrease in heparosan molecular weight and formation of an undesired double bond at the chain's non‐reducing end, it would be beneficial to include the gene for Δ‐4,5‐glycuronidase in an inducer controlled lyase expression system. This will facilitate the removal of unsaturated sugar units from the secreted heparosan by this enzyme and possibly result in more well‐defined chain lengths of the polysaccharide product.^76^ Thus, through metabolic engineering, the yield and structural quality of the heparosan product, and ultimately the heparin product, can be enhanced.

#### 3.4.3 *E. coli* K‐12

When the region 2 *E. coli* K5 heparosan biosynthesis genes *kfiABCD* are cloned and expressed into *E. coli* K‐12, the bacterial heparosan capsule can be produced as a primarily intracellular product, as long as exportation genes in other regions are suppressed. A recombinant bacterial strain was obtained by cloning the *kfi* genes in pairs (*kfiAB* and *kfiCD*) into IPTG‐inducible plasmids. Fed‐batch fermentation of this strain of co‐expressed genes in minimal media showed that the majority of product stayed inside the cells as expected, and its molecular weight (105 kD) was higher than that produced extracellularly in *E. coli* K5 (50‐80 kD) or in *E. coli* K‐12 overexpressing all three regions of the K5 cluster (65 kD). The intracellular yield of the polysaccharide product was also higher than for extracellular *E. coli* K5. Based on these results it appears that the exportation system impacts the size of the polysaccharide. Intracellular expression of heparosan lyase, an enzyme that degrades the heparosan chains into LMW polymers, combined with the region 2 biosynthesis genes in a new recombinant K‐12 strain, led to in vivo bacterial production and refinement of the heparosan hexasaccharide as a precursor for intracellular heparin synthesis.^77^

#### 3.4.4 *E. coli* BL21

Bacterial biosynthesis of heparosan has traditionally been achieved using *E. coli* K5, but this pathogenic bacterial strain can be beneficially replaced by non‐pathogenic *E. coli* BL21 as a production host. Varying expression levels of the region 2 heparosan biosynthesis genes of the K5 gene cluster in BL21, using an inducible plasmid, can result in different product yields. A medium copy plasmid was used for the expression of *kfiA/kfiC*, and a high copy plasmid was used for expressing *kfiB/kfiD* to balance the influence of GAG production on host metabolism. When this recombinant *sABCD* strain co‐expressing the four K5 biosynthesis genes was transformed into BL21, the highest yields obtained from fermentation experiments were 334 mg/L in shake flasks, 652 mg/L in a 3 L batch culture, and 1.88 g/L in a fed‐batch culture.^62^ A competitive relationship was observed between cell growth and heparosan production. These two phenomena must be carefully balanced to achieve high product yield since they both share the common precursor of glucose‐1‐phosphate, which is also used for cell wall biosynthesis. A similar correlation is observed with extracellular hyaluronic acid production competing with cell growth, where *E. coli* is unable to simultaneously support successful cell growth and synthesis of the exopolysaccharide hyaluronic acid.^77^

Although K5 and the recombinant BL21 strain both produce the same repeating disaccharide unit, the heparosan products differ in molecular weight and polydispersity. Chain elongation enzymes can be successfully expressed in the BL21 recombinant strain but it lacks the K5 gene encoding heparosan lyase, required for degrading long polymer chains into LMW ones. Another trade‐off was that overall product titers were found to be lower with the safer BL21 strain.^77^

#### 3.4.5 Bacillus subtilis

##### 3.4.5.1 Chondroitin and heparosan biosynthesis

*Bacillus subtilis* is a Gram‐positive bacterium, which has the advantage of being a well‐characterized strain that is generally regarded as safe. For the production of GAGs in particular, *B. subtilis* as a host is unlikely to produce degradative enzymes that will block the buildup of products like CS and heparin. Heparosan synthase encoding genes from *E. coli* K5, *kfiA* and *kfiC*, were cloned and inserted into the integration vector pAX01. Likewise, the *kfoA* and *kfoC* chondroitin pathway genes of *E. coli* K4 were amplified and subcloned into the same integration vector, then both of these assembled plasmids were transformed into *B. subtilis* 168 strain. Metabolic engineering and optimization of the synthetic pathways led to maximum yields of 5.82 and 5.22 g/L for heparosan and chondroitin, respectively, and the quality of the products was found to closely resemble natural animal‐derived CS and heparin. Further increases in yield could be achieved by up‐regulation of the *tuaD* gene that encodes for the UDP‐glucose dehydrogenase.^78^ The production of large amounts of the extracellular polysaccharide HA has an associated high metabolic burden and the impact of diverting required sugars and other molecules from their vital cellular roles. However, unlike in *E. coli*, expression of the *Streptococcus hasA* gene in an engineered *Bacillus* strain led to the production of HA at high levels during fermentation, without compromising the cell's capacity to grow.^78^

## 4 Potential caveats and considerations

There are several considerations that need to be addressed before bioengineered heparin can be produced in large scale. The most important of these is the optimization and standardization of the production of all the required biosynthetic enzymes. Each of these enzymes needs to be produced in large scale and with enhanced stability. Moreover, additional studies are required to more fully understand the specificity of each enzyme including their different isoforms. The cost of the in vitro chemoenzymatic production of heparin should be comparable to heparin produced from pig intestine. The in vivo preparation of heparin in metabolically engineered mast cells or CHO cells will probably not be competitive in the large‐scale production of heparin but may find a niche in the preparation of small amounts of designer heparins. The metabolic engineering of *E. coli* to produce heparin still requires the solution of enormous challenges including the active expression of NDST and the integration of enzymatic steps required for the stepwise conversion of heparosan to heparin.
